# Clinical Outcomes of Asynchronous Versus Synchronous Telepsychiatry in Primary Care: Randomized Controlled Trial

**DOI:** 10.2196/24047

**Published:** 2021-07-20

**Authors:** Peter M Yellowlees, Michelle Burke Parish, Alvaro D Gonzalez, Steven R Chan, Donald M Hilty, Byung-Kwang Yoo, J Paul Leigh, Robert M McCarron, Lorin M Scher, Andres F Sciolla, Jay Shore, Glen Xiong, Katherine M Soltero, Alice Fisher, Jeffrey R Fine, Jennifer Bannister, Ana-Maria Iosif

**Affiliations:** 1 Department of Psychiatry and Behavioral Sciences University of California Davis Sacramento, CA United States; 2 Stanford University School of Medicine Stanford, CA United States; 3 Veterans Administration Palo Alto Healthcare System Palo Alto, CA United States; 4 Northern California Veterans Administration Mather, CA United States; 5 Department of Public Health Sciences University of California Davis Davis, CA United States; 6 University of California Irvine Irvine, CA United States; 7 University of Colorado Anschutz Medical Campus Denver, CO United States; 8 CommuniCare Health Centers, West Sacramento Sacramento, CA United States

**Keywords:** asynchronous telepsychiatry, synchronous telepsychiatry, psychiatrist, primary care physician, psychiatric consultation, Spanish-speaking, collaborative care, workforce, depression, telehealth

## Abstract

**Background:**

Asynchronous telepsychiatry (ATP; delayed-time) consultations are a novel form of psychiatric consultation in primary care settings. Longitudinal studies comparing clinical outcomes for ATP with synchronous telepsychiatry (STP) are lacking.

**Objective:**

This study aims to determine the effectiveness of ATP in improving clinical outcomes in English- and Spanish-speaking primary care patients compared with STP, the telepsychiatry *usual care* method.

**Methods:**

Overall, 36 primary care physicians from 3 primary care clinics referred a heterogeneous sample of 401 treatment-seeking adult patients with nonurgent psychiatric disorders. A total of 184 (94 ATP and 90 STP) English- and Spanish-speaking participants (36/184, 19.6% Hispanic) were enrolled and randomized, and 160 (80 ATP and 80 STP) of them completed baseline evaluations. Patients were treated by their primary care physicians using a collaborative care model in consultation with the University of California Davis Health telepsychiatrists, who consulted with patients every 6 months for up to 2 years using ATP or STP. Primary outcomes (the clinician-rated Clinical Global Impressions [CGI] scale and the Global Assessment of Functioning [GAF]) and secondary outcomes (patients’ self-reported physical and mental health and depression) outcomes were assessed every 6 months.

**Results:**

For clinician-rated primary outcomes, ATP did not promote greater improvement than STP at 6-month follow-up (ATP vs STP, adjusted difference in follow-up at 6 months vs baseline differences for CGI: 0.2, 95% CI −0.2 to 0.6; *P*=.28; and GAF: −0.6, 95% CI −3.1 to 1.9; *P*=.66) or 12-month follow-up (ATP vs STP, adjusted difference in follow-up at 12 months vs baseline differences for CGI: 0.4, 95% CI −0.04 to 0.8; *P*=.07; and GAF: −0.5, 95% CI −3.3 to 2.2; *P*=.70), but patients in both arms had statistically and clinically significant improvements in both outcomes. There were no significant differences in improvement from baseline between ATP and STP on any patient self-reported ratings at any follow-up (all *P* values were between .17 and .96). Dropout rates were higher than predicted but similar between the 2 arms. Of those with baseline visits, 46.8% (75/160) did not have a follow-up at 1 year, and 72.7% (107/147) did not have a follow-up at 2 years. No serious adverse events were associated with the intervention.

**Conclusions:**

This is the first longitudinal study to demonstrate that ATP can improve clinical outcomes in English- and Spanish-speaking primary care patients. Although we did not find evidence that ATP is superior to STP in improving clinical outcomes, it is potentially a key part of stepped mental health interventions available in primary care. ATP presents a possible solution to the workforce shortage of psychiatrists and a strategy for improving existing systems of care.

**Trial Registration:**

ClinicalTrials.gov NCT02084979; https://clinicaltrials.gov/ct2/show/NCT02084979.

## Introduction

### Background

Telepsychiatry, in the form of videoconferencing, is now an important tool in behavioral health care. For more than 30 years, synchronous telepsychiatry (STP), where consultations are performed in real time and are interactive, has increased access to care, making psychiatric experts available in areas with provider shortages. Research has demonstrated high levels of patient satisfaction and similar clinical outcomes to traditional in-person care for many disorders, including depression and anxiety [1,2]. Telemedicine utilization across all disciplines had already been anticipated to grow exponentially to a 130 billion dollar industry by 2025 [3], before the use of telepsychiatry dramatically increased during the COVID-19 pandemic. Suddenly, telepsychiatry has become a core health care tool [4] for most psychiatrists in the United States. Many clinics rapidly converted to telepsychiatry, with a number of them describing the experience and the changes required including the move to in-home consultations or virtual house calls. For example, the large University of California Davis (UCD) behavioral health outpatient clinic saw a successful conversion from approximately 97% in-person consultations to 100% virtual consultations in 3 days [4]. A survey conducted by the American Psychiatric Association during the COVID-19 pandemic found that by June 2020, 85% of 500 surveyed American psychiatrists were using telepsychiatry with more than 75% of their patients, compared with about 3% before COVID-19 [5]. The latest available national telehealth statistics derived from 60 contributing private insurers to the Fair Health database [6] as of December 2020 showed an increase of 2816% in telehealth consultations in all disciplines compared with December 2019, and these comprised 6.5% of all consultations nationally in their database, with 47% of the patients being seen primarily for mental health reasons [6]. The National Center for Health Statistics [7] reported a total of 883 million outpatient consultations nationally in 2018. Projecting from the insurance statistics [6], we can assume that about 3% of these visits in 2020 were telepsychiatry visits (by video or phone), an approximate total of 26 million such visits. There seems no doubt that STP has now become a major delivery component of mental health services in the United States.

### Asynchronous Care

Despite this success, with STP being the current standard telepsychiatry practice [8,9], administrative and technical challenges exist, especially around scheduling of telepsychiatrists and patients [10,11], and STP itself is simply a virtual extension of in-person care that cannot be scaled to enable a physician to see more patients. Asynchronous care makes use of a completely virtual care model with the transmission of clinical information via web applications for review by a specialist at a later time [12,13] and has the potential to scale and enable psychiatrists to be involved in the treatment of more patients than with STP. Asynchronous care can also reduce the impact of poor bandwidth and connectivity issues seen with STP, providing potentially better access to more diverse patient populations. In recent years, asynchronous technologies have become more widespread in health care settings, utilized in patient portal email and messaging, in-app messaging, specialty patient-to-provider mobile apps, and forwarded interview videos [14]. Asynchronous technologies are commonly used in radiology, dermatology, ophthalmology, cardiology, and pathology [12,15-22] and are expanding into mental health care where they may be at least a partial solution to address the psychiatrist workforce shortage and reduce access barriers for patients [14]. Studies indicate that up to 10% of those who reported experiencing mental health difficulties are able to utilize available asynchronous resources [23], which can improve self-management and access. Positive patient outcomes have been demonstrated with e-coaching for depression [24], mobile-based asynchronous text-messaging therapy with a licensed therapist [25], integrated asynchronous virtual care platform [26], and asynchronous telehealth [27]. In addition to improved patient outcomes, such services have been found to increase access and care quality and reduce overutilization and costs of care [26].

Asynchronous telepsychiatry (ATP) is a more data-rich form of the traditional medical or psychiatric *curbside consultation*. In ATP, a trained interviewer conducts and records a semistructured patient interview, which is combined with other available clinical data, such as electronic medical records (EMRs). This recorded video consult and information from the EMR is made accessible to a telepsychiatrist who reviews it before providing an opinion on the patient’s diagnosis and treatment options. The process, including consultation templates, has been fully described in previous publications [8,28-33]. Early pilot studies provide evidence that ATP has similar diagnostic accuracy to STP in English- and Spanish-speaking patients, that it is a feasible consultation modality in primary care patients and with patients cared for in skilled nursing facilities [28-35], and that it may also be less costly to implement.

### Objectives

This paper describes the first large study conducted to determine the effectiveness of ATP in improving clinical outcomes, as compared with STP, the current gold standard telepsychiatry *usual care* treatment method. We hypothesized that, compared with participants in the STP arm, participants in the ATP arm would show better clinical trajectories throughout treatment, as measured by greater improvements in clinician and patient self-reported ratings of global functioning, health outcomes, and depression.

## Methods

### Study Design and Setting

The study was a randomized controlled clinical trial conducted at 3 community-based primary care clinics in the Sacramento area, with patient recruitment occurring between March 2014 and September 2018. A data and safety monitoring board (DSMB), consisting of 2 independent physicians and 1 statistician, periodically reviewed and evaluated the accumulated study data for participant safety, study conduct, and progress. In a DSMB review in early 2018, it was noted that the dropout rate at 24 months was higher than anticipated. Thus, the DSMB recommended that the primary end point be at 12 months and advised subsequent enrollment be limited to the 12-month follow-up. The 12-month follow-up was the primary analysis of interest, and the institutional review board (IRB) documentation was modified in April 2018. The last 18 patients were enrolled for 1 year.

### Participants

We recruited 36 primary care physicians (PCPs) as referring providers. We placed an alert in the electronic medical record system to remind the PCPs about the trial. Patients learned about the study through their PCP or from advertisements at the referring clinics. All participants were aged 18 years or older, able to give written informed consent, and were referred by PCPs as having one or more nonurgent mental health diagnoses, mainly mood disorders, anxiety disorders, or substance and alcohol use disorders. Many patients had comorbid conditions and multiple diagnoses. We attempted to overenroll Spanish-speaking patients and included a Federally Qualified Health Center that primarily treats Spanish-speaking patients as one of our referring clinics. The study protocol was approved by the UCD IRB; written informed consent was obtained from both patients and the referring PCPs before participation.

Potential participants completed a multistep screening and enrollment process. This consisted of a semistructured phone interview as well as the Patient Health Questionnaire-9 (PHQ-9) [36] to screen for risk of suicidality, followed by an in-person assessment with the Structured Clinical Interview for the Diagnostic Statistical Manual (SCID) IV Diagnoses in English or Spanish [37]. The SCID established a primary axis I diagnosis, which was used for stratified randomization. Before enrolling the first participant, the study statistician created a stratified block randomization schedule for each study site. Within each site, patients were assigned 1:1 to the 2 intervention arms in random permuted blocks of size 4 generated for each SCID primary axis I categorization to reduce imbalance between arms.

Patients were recruited for 4 study years and were followed up between 1 and 2 years with ATP or STP evaluations every 6 months (up to 5 visits). Four UCD faculty psychiatrists provided consultations for both ATP and STP groups, with a bilingual psychiatrist seeing all the patients who could only speak Spanish or preferred to have their consultations in that language. All psychiatrists were fully trained to deliver both types of consultations. Diagnostic conclusions and treatment recommendations of the consulting psychiatrist for all patients were reported back to the PCP in the psychiatrists’ notes in the EMR. The PCPs then implemented the recommendations at their own discretion and could also communicate further by secure messaging or phone with the psychiatrists if they wished. ATP interviewers in the trial were behavioral health clinicians with a master’s degree or higher, and their training for this trial has already been described [38].

### Intervention Arm

ATP assessments were conducted at 6-month intervals by an ATP-trained clinician who spoke the patient’s primary language, either English or Spanish [38]. This interview was video-recorded using Health Insurance Portability and Accountability Act–compliant security systems and protocols. For each ATP assessment, the clinician updated a standardized electronic form to capture notes about clinically relevant or important materials observed during the interview. These notes were usually completed the day of the ATP interview so that study psychiatrists had rapid access to the entire interview video, the clinician’s interview notes, and previous medical and sometimes psychiatric assessments of the patient already recorded in their EMR. After each ATP visit, the psychiatrist provided the patient’s PCP with a written assessment and psychiatric treatment plan. The PCP also had continued access to this psychiatrist by phone or email between the study consultations for up to 2 years [8,38].

### Control Arm

The clinical workflow process for the STP arm was similar to that of the ATP arm, except that ATP-recorded assessments were replaced by live real-time STP assessments conducted by a psychiatrist who spoke the patient’s preferred language, either English or Spanish. After the STP consultation, the psychiatrist provided the patient’s PCP with a written assessment and treatment plan in the patient’s EMR and was available for future contact by phone or email as necessary.

A demographic questionnaire was administered at the baseline to collect sociodemographic information. Participants were clinically assessed in both study arms at 6-month intervals (baseline, 6 months, 12 months, 18 months, and 24 months), with the primary outcome measures completed by the treating psychiatrists. All other study questionnaires assessing self-reported outcomes were collected every 6 months by research assistants either by phone or via paper or electronic surveys, depending on participants’ preferences. Participants were compensated for each assessment visit with a US $25 gift card, an amount considered by the IRB to be noncoercive. The PCPs were not compensated.

### Primary Outcomes

The primary outcomes were derived from the psychiatrist’s report and included the Clinical Global Impressions (CGIs) scale [39], which focuses primarily on functional impairment, and the Global Assessment of Functioning (GAF) [40], which mainly measures symptom severity. The CGI is a 3-item, 7-point observer-rated scale that measures illness severity, global improvement or change, and therapeutic response. The CGI is considered a robust measure with established validity in inpatient [41], outpatient [42], and clinical trial settings [42]. The CGI severity of illness and improvement scales are commonly used in nondrug trial settings [39]. We used the CGI severity of illness scale scored from 1 (normal) to 7 (among the most extremely ill). The GAF is a widely used rating scale for assessing impairment among patients with psychiatric disorders. The GAF assesses the level of psychological, social, and occupational functioning on a scale of 1 to 100, with higher levels indicating better functioning [40].

### Secondary Outcomes

Secondary outcomes focused on patient self-report and included the 12-Item Short-Form Health Survey Physical Health Summary (PHS-12) and 12-Item Short-Form Health Survey Mental Health Summary (MHS-12) [43] scores (both scored from 0-100, with higher scores indicating better health) and the PHQ-9 [44]. The PHQ-9 is a well-validated depression scale with scores derived as the sum of 9 items (each scored from 0 [not at all] to 3 [nearly every day]; scale range 0-27) based directly on the diagnostic criteria for major depressive disorder in the Diagnostic and Statistical Manual Fourth Edition [37].

### Sample Size Calculation

The statistical power to assess the difference in improvement from baseline to the primary end point (12 months) between ATP and STP for the clinician and patient-reported measures was evaluated assuming that 100 patients would be randomized into each arm, an attrition rate of 25%, and that half an SD was the smallest difference that would be clinically meaningful. Assuming a type 1 error *α*=.05, a two-sided test, measurements at baseline, 6, and 12 months, correlations between repeated measures ranging from 0.30 to 0.60, and the proposed sample size of 75 patients in each arm (after 25% loss to follow-up), we anticipated having 83%-96% power to detect half an SD difference in improvement between the arms at 12 months. On the basis of published data, we estimated a residual SD of 1.5 for CGI [45] and 10-12 points for the mental (MHS-12) and physical health (PHS-12) subscales of the 12-Item Short-Form Health Survey [46]. Thus, under the above assumptions, this study was sufficiently powered to detect a difference in improvement between the arms at 12 months of 0.75 for CGI and 5-6 points on MHS-12 and PHS-12. The calculation assumes 75 patients per arm, but some of the patients lost to follow-up would have completed some evaluations and contributed data, and thus the power would be higher. In addition, the power would be higher if the correlation between repeated measures was greater than 0.6.

### Statistical Analysis

Group differences in demographic and clinical characteristics were assessed using χ^2^ test (or Fisher exact test) for categorical variables and the two-sample (two-tailed) *t* tests (or Wilcoxon two-sample test) for continuous variables, as appropriate.

All analyses were intention-to-treat, and patients were analyzed as randomized. Mixed-effects linear regression models [47] were used to characterize the longitudinal trajectories of primary and secondary outcomes and assess intervention effects. This approach explicitly accounts for multiple measurements per person, allows for unequally spaced and missing observations, and produces valid inferences under the assumption that data are missing at random. The primary end point was the 12-month follow-up, and all participants who had at least 1 follow-up clinician rating at 6 or 12 months were included in the primary analysis. For each outcome variable, we fit a model that included terms for the intervention arm (ATP or STP), time (baseline, 6 months, and 12 months), and the interaction between arm and time. Models for clinician-rated outcomes were adjusted for a composite variable whose levels captured all possible combinations of study sites, treating psychiatrists, and language of the interview. Models for patient self-reported outcomes were similarly adjusted for a composite variable whose levels captured all possible combinations of study site, person conducting the interview (ATP interviewer or STP psychiatrist), and language of the interview. We accounted for clustering using a random effect for the patient and, whenever possible, a random effect for the referring physician. The interaction terms allowed us to assess intervention effects, that is, adjusted differences in follow-up compared with baseline differences between ATP and STP. All contrasts were estimated with 95% CIs and tested with two-sided alternatives using *P*<.05 as a threshold for statistical significance. No adjustments were made for multiple comparisons. Secondary analyses to confirm the longitudinal pattern from the primary analyses were conducted using the data up to 24 months and included all participants with at least 1 follow-up visit. Sensitivity analyses controlling for baseline values were conducted to confirm the primary analysis results. All analyses were performed using PROC MIXED in SAS version 9.4 (SAS Institute Inc) [48].

## Results

### Overview

**Figure 1:**
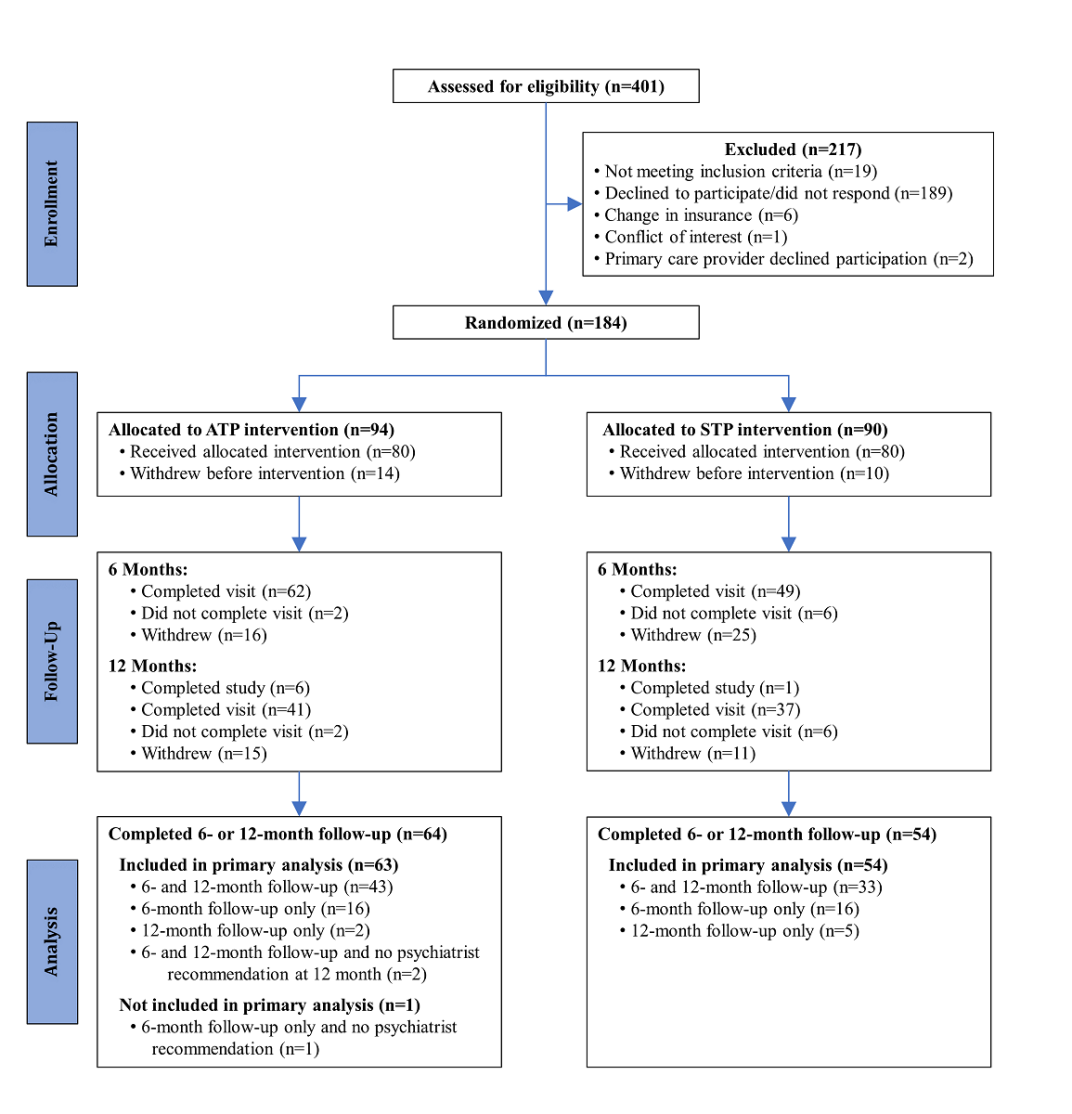
Participants flow through 12-month follow-up. ATP: asynchronous telepsychiatry; STP: synchronous telepsychiatry.

Of the 36 consented clinicians, 28 (78%) referred at least one patient. Figure 1 depicts the flow of patients from screening through the primary end point and the 12-month follow-up. Of the 401 patients assessed for eligibility, 184 (45.9%) were enrolled and randomized to the ATP (n=94) or STP (n=90) intervention. Of the 184 randomized participants, 18 (9.8%; 11 ATP and 7 STP) consented to the 12-month follow-up, and 24 (13%; 14 ATP and 10 STP) withdrew before the baseline visit. Reasons for withdrawal before baseline included insurance changes (n=2), decline to participate (n=7), and loss to follow-up (n=15). Multimedia Appendix 1, Table S1 shows the demographic and clinical characteristics of the 160 participants who completed the baseline visit and the 24 who did not. The 2 groups were similar in terms of sociodemographic characteristics and depression symptoms, but participants who completed the baseline visit were more likely to be receiving current outpatient psychotherapy for a psychiatric condition (65/158, 41.1% vs 5/24, 21%; *P*=.06) and to be using psychotropic medication (130/157, 82.8% vs 12/24, 50%; *P*<.001) than those who did not complete baseline visits. Interestingly, only 1 of these 160 patients who completed a baseline visit was seeing an outpatient psychiatrist, with the rest being treated in primary care.

Figure S1 in Multimedia Appendix 1 depicts the flow of patients from screening through the last assessment of the study (24-month follow-up). The dropout rates were higher than originally anticipated. From baseline to the end of the study, 64% (51/80) patients in the ATP arm and 78% (62/80) patients in the STP arm did not complete the study as enrolled (ie, for 12 or 24 months). In the ATP group, 9% (7/80) of the participants had insurance changes, 23% (18/80) declined to continue, 31% (25/80) were lost to follow-up, and 1 did not complete the study because of an administrative scheduling error. In the STP group, 14% (11/80) had insurance changes, 18% (14/80) declined to continue, 43% (34/80) were lost to follow-up, 1 participant was too ill to continue the study, and 2 participants died. There was no association between arm assignment and the reasons for not completing the study (*P*=.33).

Baseline demographic and clinical characteristics by study arm are presented in Table 1. There were no significant arm differences in any of these characteristics, indicating that the randomization was effective. Of the 160 participants, 137 (85.6%) were White and 30 (18.8%) were Hispanic. Most patients (54 ATP, 54 STP, ie, 67% for each arm) had depressive disorders as their primary diagnosis. Of the 160 patients with baseline data, 117 (73.1%) completed follow-up at 6 months or 12 months and had usable primary outcome data (CGI or GAF) for at least 1 follow-up and were included in the primary analyses (Figure 1). Multimedia Appendix 1, Table S2 compares the demographic and clinical characteristics of the participants who completed the baseline visit and were included (n=117) or excluded (n=43) from the primary analyses. Participants excluded from the primary analyses were more likely to have been patients in the Auburn clinic and had lower CGI scores and higher GAF scores at baseline (Multimedia Appendix 1, Table S2).

**Table 1:** Baseline demographic and clinical characteristics of participants who completed baseline visits^a^.

Characteristics^b^			ATP^c^ (n=80)		STP^d^ (n=80)		Total (n=160)
Age (years), mean (SD)			53 (14)		52.2 (14.6)		52.6 (14.3)
Number of axis I diagnoses, mean (SD)			2.4 (1)		2.4 (1)		2.4 (1)
Screening PHQ-9^e^ score^f,g^, mean (SD)			13.9 (6.6)		13.1 (5.9)		13.5 (6.3)
**Screening PHQ-9 category^g^, n (%)**							
	0-4, nondepressed	5 (6.3)		8 (10.3)		13 (8.3)	
	5-9, mild depression	18 (22.8)		16 (20.5)		34 (21.7)	
	10-14, moderate depression	21 (26.6)		23 (29.5)		44 (28)	
	≥15, moderately severe to severe depression	35 (44.3)		31 (39.7)		66 (42)	
**Primary diagnosis, n (%)**							
	Mood disorder	54 (67.5)		54 (67.5)		108 (67.5)	
	Anxiety disorder	16 (20)		16 (20)		32 (20)	
	Substance abuse	2 (2.5)		1 (1.3)		3 (1.9)	
	Other	8 (10)		9 (11.3)		17 (10.6)	
Female, n (%)			58 (72.5)		53 (66.3)		111 (69.4)
Hispanic ethnicity, n (%)			15 (18.8)		15 (18.8)		30 (18.8)
**Education, n (%)**							
	Graduate high school or less	22 (27.5)		18 (22.5)		40 (25)	
	Some college or 2-year college	32 (40)		40 (50)		72 (45)	
	College or graduate school	26 (32.5)		22 (27.5)		48 (30)	
**Marital status^h^, n (%)**							
	Married or living with someone	39 (51.3)		39 (52.7)		78 (52)	
	Other^i^	37 (48.7)		35 (47.3)		72 (48)	
Current psychiatric treatment^j^, n (%)			31 (39.7)		34 (42.5)		65 (41.1)
Current psychotropic medication^k^, n (%)			64 (83.1)		66 (82.5)		130 (82.8)
**Language of the interview, n (%)**							
	English	71 (88.8)		70 (87.5)		141 (88.1)	
	Spanish	9 (11.3)		10 (12.5)		19 (11.9)	
**Study clinic, n (%)**							
	Auburn	44 (55)		43 (53.8)		87 (54.4)	
	J Street (Sacramento)	17 (21.3)		19 (23.8)		36 (22.5)	
	Communicare	19 (23.8)		18 (22.5)		37 (23.1)	

^a^Due to rounding, percentages might not sum to 100.

^b^There were no significant differences between the 2 intervention groups for any characteristic.

^c^ATP: asynchronous telepsychiatry.

^d^STP: synchronous telepsychiatry.

^e^PHQ-9: Patient Health Questionnaire-9.

^f^Range 0-27, higher is more depressed.

^g^Data missing=1 in asynchronous telepsychiatry group and 2 in synchronous telepsychiatry.

^h^Data missing=4 in asynchronous telepsychiatry group and 6 in synchronous telepsychiatry.

^i^Includes widowed, divorced or annulled, separated, and never married.

^j^Data missing=2 in asynchronous telepsychiatry group.

^k^Data missing=3 in asynchronous telepsychiatry group.

### Primary Outcomes

Table 2 summarizes the mean trajectories and changes from baseline in the 2 intervention arms for the clinician ratings (CGI and GAF) and the results of mixed-effects models for the primary analysis. For both CGI and GAF, ATP did not promote greater improvement than STP at the 6- (ATP vs STP, adjusted difference in follow-up at 6 months vs baseline differences for CGI: 0.2, 95% CI −0.2 to 0.6; *P*=.28; and GAF: −0.6, 95% CI −3.1 to 1.9; *P*=.66) or 12-month follow-up (ATP vs STP, adjusted difference in follow-up at 12 months vs baseline differences for CGI: 0.4, 95% CI −0.04 to 0.8; *P*=.07; and GAF: −0.5, 95% CI −3.3 to 2.2; *P*=.70). However, both the ATP and STP arms improved at 6 and 12 months compared with baseline. Patients in both arms had about 1 point improvement in CGI at 6-month follow-up (estimated difference from baseline −0.7, 95% CI −1.0 to −0.4; *P*<.001 for ATP; and −0.9, 95% CI −1.2 to −0.6; *P*<.001 for STP), and these improvements were maintained at 12 months (estimated difference from baseline −0.8, 95% CI −1.1 to −0.5; *P*<.001 for ATP; and −1.2, 95% CI −1.5 to −0.9; *P*<.001 for STP). The results for GAF were similar, with both groups improving by about 3 points at 6-month (estimated difference from baseline 2.7, 95% CI 1.1-4.4; *P*=.002 for ATP; and 3.3, 95% CI 1.4-5.1; *P*<.001 for STP) and by about 5 points at 12-month follow-up (estimated difference from baseline 4.7, 95% CI 2.8-6.5; *P*<.001 for ATP; and 5.2, 95% CI 3.2-7.2; *P*<.001 for STP).

**Table 2:** Primary outcomes: clinician ratings at baseline and 6- and 12-month follow-up for the 117 patients included in the primary analysis.

Primary outcomes				Patient, n (%)		CGI^a^, mean (SD)		GAF^b^, mean (SD)		CGI; estimate, mean (95% CI)^c^		GAF; estimate, mean (95% CI)^c^
**ATP^d^**												
	**Mean trajectory**											
		Baseline	63 (100)		3.9 (0.9)		59.7 (10.8)		—^e^		—	
		Follow-up at 6 months	61 (97)		3.2 (1)		62.4 (11.9)		—		—	
		Follow-up at 12 months	45 (71)		3.1 (1.1)		63.7 (13)		—		—	
	**Change from baseline**											
		6 months versus baseline	61 (97)		−0.7 (1)		2.8 (6.3)		−0.7 (−1.0 to −0.4)		2.7 (1.1 to 4.4)	
		12 months versus baseline	45 (71)		−0.8 (1.2)		4.4 (8.7)		−0.8 (−1.1 to −0.5)		4.7 (2.8 to 6.5)	
**STP^f^**												
	**Mean trajectory**											
		Baseline	54 (100)		4.2 (1)		57.6 (10.2)		—		—	
		Follow-up at 6 months	49 (91)		3.3 (1)		60.7 (11.0)		—		—	
		Follow-up at 12 months	38 (70)		3.0 (1)		61.8 (12.2)		—		—	
	**Change from baseline**											
		6 months versus baseline	49 (91)		−0.9 (1)		2.9 (6.4)		−0.9 (−1.2 to −0.6)		3.3 (1.4 to 5.1)	
		12 months versus baseline	38 (70)		−1.2 (1)		5.1 (6.3)		−1.2 (−1.5 to −0.9)		5.2 (3.2 to 7.2)	
ATP versus STP, difference at baseline				—		—		—		−0.3 (−0.6 to 0.1)		0.9 (−2.1 to 4)
ATP versus STP, difference at follow-up at 6 months				—		—		—		−0.1 (−0.4 to 0.3)		0.4 (−2.8 to 3.5)
ATP versus STP, difference at follow-up at 12 months				—		—		—		0.1 (−0.3 to 0.5)		0.4 (−2.9 to 3.7)
ATP versus STP, difference in follow-up at 6 months versus baseline differences				—		—		—		0.2 (−0.2 to 0.6)		−0.6 (−3.1 to 1.9)
ATP versus STP, difference in follow-up at 12 months versus baseline differences				—		—		—		0.4 (−0.04 to 0.8)		−0.5 (−3.3 to 2.2)

^a^CGI: Clinical Global Impression scale; severity of illness; range 1-7, higher is more severe.

^b^GAF: Global Assessment of Functioning; range 0-100, higher is better functioning.

^c^From mixed-effects linear regression models adjusted for study site, consulting psychiatrist, and language of the interview, as well as clustering due to patient. The model for the Global Assessment of Functioning was further adjusted for clustering by the referring physician.

^d^ATP: asynchronous telepsychiatry.

^e^Not available.

^f^STP: synchronous telepsychiatry.

### Secondary Outcomes

Tables 3 and 4 show the descriptive statistics and the results of mixed-effects models for patient self-reported ratings: PHS-12, MHS-12, and PHQ-9, respectively. The pattern of the self-reported ratings was less consistent throughout the follow-up for both ATP and STP arms, with only the mental health score in STP showing statistically significant improvement at 6 months and the PHQ-9 score showing improvement in the ATP group at both 6 and 12 months. However, there were no statistically significant differences in improvement between the intervention arms at any time point for any patient self-reported ratings.

**Table 3:** Secondary outcomes: patient self-reported 12-Item Short-Form Health Survey (physical and mental) scores at baseline and 6- and 12-month follow-up for the 117 patients included in the primary analysis.

Secondary outcomes				Patient, n (%)		PHS-12^a^, mean (SD)		MHS-12^b^, mean (SD)		PHS-12; estimate, mean (95% CI)^c^		MHS-12; estimate, mean (95% CI)^c^
**ATP^d^**												
	**Mean trajectory**											
		Baseline	52 (83)		39.6 (11.6)		34.4 (9.6)		—^e^		—	
		Follow-up at 6 months	51 (81)		39.5 (11.5)		36.7 (9.8)		—		—	
		Follow-up at 12 months	42 (67)		38.7 (11.5)		38.2 (9.1)		—		—	
	**Change from baseline**											
		6 months versus baseline	43 (68)		−1.4 (8.8)		2 (11.9)		−1.2 (−3.9 to 11.61.6)		2.5 (−0.7 to 5.7)	
		12 months versus baseline	33 (52)		0.3 (9.3)		3.7 (12.5)		0.1 (−3.0 to 3.2)		3.6 (−0.003 to 7.1)	
**STP^f^**												
	**Mean trajectory**											
		Baseline	45 (83)		43.4 (10.4)		31.7 (8.9)		—		—	
		Follow-up at 6 months	41 (76)		41.3 (10.5)		36 (11.1)		—		—	
		Follow-up at 12 months	28 (52)		43.9 (9.4)		34.3 (10.4)		—		—	
	**Change from baseline**											
		6 months versus baseline	34 (63)		−1.8 (11.4)		5.1 (10.4)		−2.1 (−5 to 0.8)		4.7 (1.4 to 8.1)	
		12 months versus baseline	24 (44)		−1.1 (8.9)		5 (9.9)		0.001 (−3.3 to 3.3)		3.7 (-0.2 to 7.5)	
ATP versus STP, difference at baseline				—		—		—		−9.5 (−32.5 to 13.6)		−2.7 (−24.1 to 18.8)
ATP versus STP, difference at follow-up at 6 months				—		—		—		−8.6 (−31.5 to 14.4)		−4.9 (−26.1 to 16.3)
ATP versus STP, difference at follow-up at 12 months				—		—		—		−9.4 (−32.5 to 13.8)		−2.8 (−24.4 to 18.8)
ATP versus STP, difference in follow-up at 6 months versus baseline differences				—		—		—		0.9 (−3.1 to 4.9)		−2.2 (−6.9 to 2.5)
ATP versus STP, difference in follow-up at 12 months versus baseline differences				—		—		—		0.1 (−4.4 to 4.7)		−0.1 (−5.3 to 5.1)

^a^PHS-12: 12-Item Short-Form Health Survey Physical Health Summary; range 0-100, higher is better physical health.

^b^MHS-12: 12-Item Short-Form Health Survey Mental Health Summary; range 0-100, higher is better mental health.

^c^From mixed-effects linear regression models adjusted for study site, consulting psychiatrist, and language of the interview as well as clustering due to patient and primary care physician.

^d^ATP: asynchronous telepsychiatry.

^e^Not available.

^f^STP: synchronous telepsychiatry.

**Table 4:** Secondary outcomes: patient self-reported Patient Health Questionnaire-9 scores at baseline and 6- and 12-month follow-up for the 117 patients included in the primary analysis.

Secondary outcomes			Patient, n (%)	PHQ-9^a^, mean (SD)	PHQ-9; estimate, mean (95% CI)^b^
**ATP^c^**					
	**Mean trajectory**				
		Baseline	61 (97)	12.4 (7.2)	—^d^
		Follow-up at 6 months	57 (90)	9.8 (6.7)	—
		Follow-up at 12 months	45 (71)	10 (6)	—
	**Change from baseline**				
		6 months versus baseline	55 (87)	−2.3 (4.4)	−2.4 (−3.8 to −0.9)
		12 months versus baseline	43 (68)	−2.8 (5.2)	−2.2 (−3.9 to −0.5)
**STP^e^**					
	**Mean trajectory**				
		Baseline	53 (98)	12.6 (6.8)	—
		Follow-up at 6 months	40 (74)	10.8 (6.5)	—
		Follow-up at 12 months	34 (63)	11.9 (7.1)	—
	**Change from baseline**				
		6 months versus baseline	40 (74)	−0.7 (4.8)	−0.9 (−2.5 to 0.8)
		12 months versus baseline	33 (61)	−0.5 (6.4)	−0.7 (−2.4 to 1.0)
ATP versus STP, difference at baseline			—	—	1.8 (−9.4 to 13.1)
ATP versus STP, difference at follow-up at 6 months			—	—	0.3 (−10.9 to 11.6)
ATP versus STP, difference at follow-up at 12 months			—	—	0.3 (−11 to 11.6)
ATP versus STP, difference in follow-up at 6 months versus baseline differences			—	—	−1.5 (−3.7 to 0.6)
ATP versus STP, difference in follow-up at 12 months versus baseline differences			—	—	−1.5 (−3.9 to 0.9)

^a^PHQ-9: Patient Health Questionnaire-9; range 0-27, higher is more depressed.

^b^From mixed-effects linear regression models adjusted for study site, consulting psychiatrist, and language of the interview, as well as clustering due to patient and primary care physician.

^c^ATP: asynchronous telepsychiatry.

^d^Not available.

^e^STP: synchronous telepsychiatry.

The results of the secondary analysis (Multimedia Appendix 1, Tables S3-S5) parallel those of the primary analysis, with ATP and STP groups maintaining improvements in both CGI and GAF at 18 and 24 months as compared with baseline and showed no significant interactions between the intervention arm and follow-up times. Sensitivity analyses adjusted for baseline score severity confirmed the results of the primary analyses.

### Treatment Adherence, Data Availability, and Unanticipated Events

Adverse or unanticipated events during the trial were reported to the IRB and the DSMB and included 2 patient deaths from unrelated medical complications and 2 patients who threatened self-harm. Both patients who threatened self-harm were urgently contacted by study psychiatrists to make clinical decisions on their follow-up care. The DSMB determined that neither event was study related. A total of 2 participants were randomized to ATP but completed the STP procedures after the baseline. One of these patients, who was urgently seen in person because of suicidal ideation, requested to continue seeing a psychiatrist through STP and was switched from ATP to STP. Another patient was misscheduled from ATP to STP and continued the study in the STP group. During the course of the study, for administrative reasons, the study psychiatrists failed to return notes for 10 completed visits (8 ATP and 2 STP).

## Discussion

### Principal Findings

This study is the first randomized controlled clinical trial to compare STP with ATP in primary care. It has a number of strengths, including being conducted in primary care practice settings using a collaborative care model (3 sites, including 1 rural site) with a diverse patient sample of English- and Spanish-speaking patients and a much longer follow-up period than most psychiatric clinical trials. Clinical outcomes were assessed using both clinician and patient self-reported ratings and included both mental health as well as broader measures of health status. A large number of PCPs continued to refer patients to the study for 4 years. The referred patients were typically individuals with mild to moderate anxiety and depression who were mainly treated by PCPs and often did not receive care from a psychiatrist.

At both 12 and 24 months of follow-up, we found that ATP was not superior to STP in improving patient outcomes. However, both ATP and STP patients showed improvements from baseline in 2 separate clinician-rated outcomes at 12-month (of about 1 point for functional impairment on the CGI and 5 points on symptom severity for the GAF) and 24-month follow-up (of about 1 point for CGI and 8 points for GAF). The magnitude of these improvements is similar to those found in recent clinical trials on the effect of nonpharmacological interventions on patient outcomes [45,49,50]. Studies suggest that the minimum clinically meaningful change on the CGI is a 1-point change [49,50], and we have not found similar studies using the CGI outcome for follow-up periods longer than 6 months. A 1-point improvement in our relatively mildly ill population, as we found, is arguably even more clinically significant than in a population that was more severely ill on average at baseline. The findings of an improvement of 8 points on the GAF are similar to long-term therapies in comparable clinical trials [51].

This study was not a noninferiority trial; the sponsor hoped to demonstrate the superiority of ATP. The results did not support the primary hypothesis that ATP promotes more improvement than STP. For clinician ratings, both the ATP and STP arms improved at similar rates throughout the trial, with no significant differences in improvements between the 2 arms. Although not supporting our primary hypothesis, this is still a clinically important result. The standardized implementation of ATP across several primary care settings for a long follow-up period, with improved clinical outcomes at 1 year and 2 years, supports the feasibility of the ATP model of care to treat depression and anxiety using a psychiatric consultation model in patients treated in primary care. This treatment option may be particularly important after the COVID-19 pandemic.

The mental health care system has been significantly affected by the COVID-19 pandemic, with what has been described as a follow-on mental health pandemic [52]. Both the World Health Organization [53] and the Centers for Disease Control [54] have published reports describing greater community levels of depression, anxiety, substance use, domestic violence, sexual abuse and related trauma, and likely suicides. Mental health professionals are required to develop new telepsychiatry protocols and digital systems to help patients who stay at home [55], whereas the number of STP consultations nationwide has dramatically escalated. ATP can provide an innovative solution to treat people in their homes as part of the COVID-19 pandemic response, and the ATP collaborative care model leverages the expertise of psychiatrists to oversee the treatment of larger numbers of patients. In 2020, we continued testing ATP methods to treat patients in their homes and nursing homes. We plan to conduct an ATP homecare trial treating psychiatric disorders in patients who have been severely affected by COVID-19.

The results of this trial have several other implications beyond COVID-19 for broadening access to psychiatric care within underserved populations and across different countries and language groups as well as in reaching new care settings.

First, the results establish that this type of consultation is worth considering as a care option in any collaborative care program. We believe that this large trial of patients treated with ATP for up to 2 years provides evidence that should enable insurers and payers to support payment for ATP consultations. We are already conducting a similar study in skilled nursing facilities, and early engagement and feedback is positive [34]. We see many more opportunities for ATP consultations for assessment and monitoring to occur not only in primary care and remotely in patients’ homes but also in pediatric and geriatric psychiatry and correctional environments and for a range of specific psychiatric assessments, such as before bariatric or transplant surgery.

Second, 19.6% (36/184) of the patients enrolled in this trial were Hispanic. It is evident that ATP with a Spanish-speaking interviewer can improve access to psychiatric care for patients who speak only Spanish. This language-matching option provides an important opportunity to increase the availability of mental health care for patients from many language groups within the United States and around the world. We are developing automated language transcription and translation systems to enable this option.

Third, ATP consultations should improve access to psychiatrists. Psychiatrists are in short supply, especially in the primary care environment, and ATP consultations for monitoring and treating patients is an approach to diminishing the impending shortage of these specialists. The 4 treating psychiatrists in this trial did most of their consultations from their usual office environment and were often able to complete their ATP consultations in *downtime* when other patients had canceled. The psychiatrists saw this as a major practical advantage of ATP, as it increased their work efficiency while guiding PCPs more quickly than typical in-person or STP consultations. We are currently undertaking an economic evaluation of trial results. Additional data from this trial will be used to evaluate patient and provider satisfaction, cost-effectiveness, and PCP adherence with the psychiatrist’s recommendations and investigate diagnosis-specific clinical outcomes in future publications.

### Limitations

There were some limitations to this study. We anticipated a 25% dropout rate for the 2-year study. However, of the 160 patients who completed baseline, 67 (41.9%; 31 ATP and 36 STP) withdrew from the study at either 6 or 12 months, and only 47 (29.4%) patients completed the study as enrolled, despite regular communication from the research team. This is not unusual, as dropout rates in long-term randomized trials for depression in primary care range from 25% to 52% at 1 year [56] and higher for longer studies [57]. Although our dropout rate was higher than initially anticipated, it was comparable with or even less than that of other similar longitudinal trials. In a study, only 43.9% of participants had data collected at 12 months [54], whereas our study retained 58.1% of participants (93/160) at 12 months. In another study, 51% of participants completed their 12-month checkup, and 19.6% dropped out of the intervention group with less than 4 weeks of participation [55]. Of the 41.9% (67/160) of patients who completed baseline interviews and withdrew from our study in the first year, some patients reported that they dropped out because they felt good and needed no further treatment, and others because they saw no improvement. Others dropped out because they moved, whereas cessation of coverage by an insurer midway through the trial forced a number of patients (n*=*18) to seek care elsewhere. Patients were recruited from primary care in Northern California, primarily experiencing depression or mood disorders. Although this population is very socially and ethnically diverse, with more than 100 languages spoken in the Sacramento region, the generalizability of our findings to other settings and types of patients is unknown. Due to the nature of the intervention, blinding for either patients or clinicians was not feasible. Finally, relatively high dropout rates in both arms may have skewed follow-up outcomes if there is a relationship between the propensity of a data point to be missing and its values, although it is difficult to predict the direction of the bias.

### Conclusions

Although this clinical trial with a 2-year follow-up period does not provide evidence for the superiority of ATP in improving clinical outcomes in comparison with STP, there was a significant improvement in primary outcomes in patients treated with either ATP or STP. Both ATP and STP promise to be important components of collaborative care systems that can increase access to psychiatrists; while ATP, because of its scalability, can improve the efficiency of psychiatric care and help alleviate the shortage of psychiatrists. The bilingual utility of ATP also shows its potential to reach non-English-speaking populations in the United States. Further research could examine the effectiveness of ATP with additional populations, settings, and cost considerations.

## Appendix

Multimedia Appendix 1 Additional data and materials.

Multimedia Appendix 2 CONSORT-eHEALTH checklist (V 1.6.1).

## Abbreviations

ATP: asynchronous telepsychiatry
CGI: Clinical Global Impression
DSMB: data and safety monitoring board
EMR: electronic medical records
GAF: Global Assessment of Functioning
IRB: institutional review board
MHS-12: 12-Item Short-Form Health Survey Mental Health Summary
PCP: primary care physician
PHQ-9: Patient Health Questionnaire-9
PHS-12: 12-Item Short-Form Health Survey Physical Health Summary
SCID: Structured Clinical Interview for the Diagnostic Statistical Manual
STP: synchronous telepsychiatry
UCD: University of California Davis
