# Supplementary material for: Clinical Outcomes of Asynchronous Versus Synchronous Telepsychiatry in Primary Care: Randomized Controlled Trial
Source: J Med Internet Res. 2021 Jul 20;23(7):e24047. doi: 10.2196/24047 (PMC8335606; doi:10.2196/24047)

**Multimedia Appendix 1.** Supplemental tables and figures.

**Table S1.** Comparison of demographic and clinical characteristics of the randomized participants by baseline visit completion status.

**Table S2.** Comparison of demographic and clinical characteristics of the participants who completed baseline and were included or excluded from the primary analysis.

# Table S3. Primary outcomes: clinician ratings at baseline and 6-, 12-, 18-, and 24-month follow-up for the 118 patients included in the secondary analysis.

# Table S4. Secondary outcomes: patient self-reported 12-Item Short Form Health Survey (physical and mental) scores at baseline and 6-, 12-, 18-, and 24-month follow-up for the 118 patients included in the secondary analysis.

# Table S5. Secondary outcomes: patient self-reported Patient Health Questionnaire-9 scores at baseline and 6-, 12-, 18-, and 24-month follow-up for the 118 patients included in the secondary analysis.

# Figure S1. Participants flow through 24-month follow-up. ATP: asynchronous telepsychiatry; STP: synchronous telepsychiatry.

**Table S1.** Comparison of demographic and clinical characteristics of the randomized participants by baseline visit completion status^a^.

| **Characteristics** | **Completed Baseline**  **(n=160)** | | | **Did not Complete Baseline**  **(n=24)** | ***P* value^b^** |
| --- | --- | --- | --- | --- | --- |
| Age (years), mean (SD) | 52.6 (14.3) | | | 49.1 (16.2) | .35 |
| Number of axis I diagnoses, mean (SD) | 2.4 (1.0) | | | 2.4 (1.0) | .96 |
| Screening PHQ-9 score^c,d^, mean (SD) | 13.5 (6.3) | | | 12.9 (7.3) | .88 |
| **Screening PHQ-9 category^d^, n (%)** |  | | |  | .23 |
| 0-4, nondepressed | 13 (8.3) | | | 4 (17.4) |  |
| 5-9, mild depression | 34 (21.7) | | | 4 (17.4) |  |
| 10-14, moderate depression | 44 (28.0) | | | 3 (13.0) |  |
| ≥15, moderately severe to severe depression | 66 (42.0) | | | 12 (52.2) |  |
| **Primary diagnosis, n (%)** | | | | | .12 |
| Mood disorder | 108 (67.5) | | | 12 (50.0) |  |
| Anxiety disorder | 32 (20.0) | | | 7 (29.2) |  |
| Substance abuse | 3 (1.9) | | | 2 (8.3) |  |
| Other | 17 (10.6) | | | 3 (12.5) |  |
| Female, n (%) | 111 (69.4) | | | 19 (79.2) | .33 |
| **Race, n (%)** | |  |  | | .76 |
| African-American | 3 (1.9) | | | 0 (0.0) |  |
| Asian | 2 (1.3) | | | 0 (0.0) |  |
| White | 137 (85.6) | | | 20 (83.3) |  |
| Other | 18 (11.3) | | | 4 (16.7) |  |
| Hispanic ethnicity, n (%) | 30 (18.8) | | | 6 (25.0) | .47 |
| **Education, n (%)** |  | | |  | 1.00 |
| Graduate high school or less | 40 (25.0) | | | 6 (25.0) |  |
| Some college/2-year college | 72 (45.0) | | | 11 (45.8) |  |
| College/graduate school | 48 (30.0) | | | 7 (29.2) |  |
| **Marital status^e^, n (%)** |  | | |  | .23 |
| Married/living with someone | 78 (52.0) | | | 9 (69.2) |  |
| Other^f^ | 72 (48.0) | | | 4 (30.8) |  |
| Current psychiatric treatment^g^, n (%) | 65 (41.1) | | | 5 (20.8) | .06 |
| Current psychotropic medication^h^, n (%) | 130 (82.8) | | | 12 (50.0) | <.001 |
| **Language of the interview, n (%)** |  | | |  | .93 |
| English | 141 (88.1) | | | 21 (87.5) |  |
| Spanish | 19 (11.9) | | | 3 (12.5) |  |
| **Study clinic, n (%)** |  | | |  | .67 |
| Auburn | 87 (54.4) | | | 13 (54.2) |  |
| J Street (Sacramento) | 36 (22.5) | | | 7 (29.2) |  |
| Communicare | 37 (23.1) | | | 4 (16.7) |  |
| **Study Arm, n (%)** |  | | |  | .45 |
| Asynchronous telepsychiatry | 80 (50.0) | | | 14 (58.3) |  |
| Synchronous telepsychiatry | 80 (50.0) | | | 10 (41.7) |  |

^a^Due to rounding, percentages might not sum to 100.

^b^*P* values from Wilcoxon two-sample tests for continuous variables, Fisher’s exact tests for screening PHQ-9 category, primary diagnosis, and race, and χ^2^ tests for all the other categorical variables.

^c^PHQ-9: Patient Health Questionnaire-9; range 0-27, higher is more depressed.

^d^Data missing=3 in Completers group and 1 in Non-completers.

^e^Data missing=10 in Completers group and 11 in Non-completers.

^f^Includes widowed, divorced or annulled, separated, and never married.

^g^Data missing=2 in Completers group.

^h^Data missing=3 in Completers group.

**Table S2.** Comparison of demographic and clinical characteristics of the participants who completed baseline and were included or excluded from the primary analysis^a^.

| **Characteristics** | **Included in the Primary Analysis**  **(n=117)** | | | **Excluded from**  **the Primary Analysis**  **(n=43)** | ***P* value^b^** |
| --- | --- | --- | --- | --- | --- |
| Age (years), mean (SD) | 51.7 (13.9) | | | 55.1 (15.3) | .22 |
| Number of axis I diagnoses, mean (SD) | 2.4 (1.1) | | | 2.3 (0.8) | .90 |
| Screening PHQ-9 score^c,d^, mean (SD) | 13.8 (6.6) | | | 12.7 (5.3) | .38 |
| **Screening PHQ-9 category^d^, n (%)** |  | | |  | .92 |
| 0-4, nondepressed | 9 (7.8) | | | 4 (9.8) |  |
| 5-9, mild depression | 24 (20.7) | | | 10 (24.4) |  |
| 10-14, moderate depression | 33 (28.4) | | | 11 (26.8) |  |
| ≥15, moderately severe to severe depression | 50 (43.1) | | | 16 (39.0) |  |
| **Baseline measurements** |  | | |  |  |
| CGI^e^, mean (SD) | 4.0 (0.9) | | | 3.7 (0.9) | .04 |
| GAF^f^, mean (SD) | 58.7 (10.6) | | | 61.2 (6.2) | .07 |
| PHQ-9^g^, mean (SD) | 12.5 (7.0) | | | 12.2 (6.1) | .86 |
| SF-12 PHS^h,i^, mean (SD) | 41.4 (11.2) | | | 42.9 (12.3) | .59 |
| SF-12 MHS^i,j^, mean (SD) | 33.1 (9.3) | | | 34.0 (10.5) | .83 |
| **Primary diagnosis, n (%) 0.54** |  | | | | .55 |
| Mood disorder | 76 (65.0) | | | 32 (74.4) |  |
| Anxiety disorder | 26 (22.2) | | | 6 (14.0) |  |
| Substance abuse | 3 (2.6) | | | 0 (0.0) |  |
| Other | 12 (10.3) | | | 5 (11.6) |  |
| Female, n (%) | 80 (68.4) | | | 31 (72.1) | .65 |
| **Race, n (%)** |  |  |  | | .78 |
| African-American | 2 (1.7) | | | 1 (2.3) |  |
| Asian | 1 (0.9) | | | 1 (2.3) |  |
| White | 101 (86.3) | | | 36 (83.7) |  |
| Other | 13 (11.1) | | | 5 (11.6) |  |
| Hispanic ethnicity, n (%) | 23 (19.7) | | | 7 (16.3) | .63 |
| **Education, n (%)** |  | | |  | .90 |
| Graduate high school or less | 30 (25.6) | | | 10 (23.3) |  |
| Some college/2-year college | 53 (45.3) | | | 19 (44.2) |  |
| College/graduate school | 34 (29.1) | | | 14 (32.6) |  |
| **Marital status^k^, n (%)** |  | | |  | .79 |
| Married/living with someone | 57 (51.4) | | | 21 (53.8) |  |
| Other^l^ | 54 (48.6) | | | 18 (46.2) |  |
| Current psychiatric treatment^m^, n (%) | 49 (42.2) | | | 16 (38.1) | .64 |
| Current psychotropic medication^n^, n (%) | 97 (84.3) | | | 33 (78.6) | .40 |
| **Language of the interview, n (%)** |  | | |  | .54 |
| English | 102 (87.2) | | | 39 (90.7) |  |
| Spanish | 15 (12.8) | | | 4 (9.3) |  |
| **Study clinic, n (%)** |  | | |  | .003 |
| Auburn | 54 (46.2) | | | 33 (76.7) |  |
| J Street (Sacramento) | 31 (26.5) | | | 5 (11.6) |  |
| Communicare | 32 (27.4) | | | 5 (11.6) |  |
| **Study Arm, n (%)** |  | | |  | .11 |
| Asynchronous telepsychiatry | 63 (53.8) | | | 17 (39.5) |  |
| Synchronous telepsychiatry | 54 (46.2) | | | 26 (60.5) |  |

^a^Due to rounding, percentages might not sum to 100.

^b^*P* values from Wilcoxon two-sample tests for continuous variables, Fisher’s exact tests for primary diagnosis and race, and χ^2^ tests for all the other categorical variables.

^c^PHQ-9: Patient Health Questionnaire-9; range 0-27, higher is more depressed.

^d^Data missing=1 for those in the primary analysis and 2 for those not included in the primary analysis.

^e^CGI: Clinical Global Impression scale; severity of illness; range 1 to 7, higher is more severe.

^f^GAF: Global assessment of functioning; range 0 to 100, higher is better functioning.

^g^Data missing=3 for those in the primary analysis and 1 for those not included in the primary analysis.

^h^PHS-12: 12-item short form health survey physical health summary score; range 0 to 100, higher is better health.

^i^Data missing=20 for those in the primary analysis and 10 for those not included in the primary analysis.

^j^MHS-12: 12-item short form health survey mental health summary score; range 0 to 100, higher is better health.

^k^Data missing=6 for those in the primary analysis and 4 for those not included in the primary analysis.

^l^Includes widowed, divorced or annulled, separated, and never married.

^m^Data missing=1 for those in the primary analysis and 1 for those not included in the primary analysis.

^n^Data missing=2 for those in the primary analysis and 1 for those not included in the primary analysis.

# Table S3. Primary outcomes: clinician ratings at baseline and 6-, 12-, 18-, and 24-month follow-up for the 118 patients included in the secondary analysis.

| **Primary outcomes** | **n** | **CGI^a^,**  **mean (SD)** | **GAF^b^,**  **mean (SD)** | **CGI; estimate,**  **mean (95% CI)^c^** | **GAF; estimate,**  **mean (95% CI)^c^** |
| --- | --- | --- | --- | --- | --- |
| **ATP^d^** |  |  |  |  |  |
| **Mean trajectory** |  |  |  |  |  |
| Baseline | 63 | 3.9 (0.9) | 59.7 (10.8) | - | - |
| Follow-up at 6 months | 61 | 3.2 (1.0) | 62.4 (11.9) | - | - |
| Follow-up at 12 months | 45 | 3.1 (1.1) | 63.7 (13.0) | - | - |
| Follow-up at 18 months | 26 | 2.6 (0.9) | 71.0 (10.2) | - | - |
| Follow-up at 24 months | 20 | 2.7 (1.0) | 71.2 (9.8) | - | - |
| **Change from baseline** Change from baseline |  |  |  |  |  |
| 6 months vs baseline | 61 | -0.7 (1.0) | 2.8 (6.3) | -0.7 (-1.0 to -0.4) | 2.7 (1.0 to 4.4) |
| 12 months vs baseline | 45 | -0.8 (1.2) | 4.4 (8.7) | -0.8 (-1.1 to -0.5) | 4.7 (2.8 to 6.7) |
| 18 months vs baseline | 26 | -1.2 (1.2) | 8.6 (9.9) | -1.1 (-1.5 to -0.8) | 7.4 (5.1 to 9.8) |
| 24 months vs baseline | 20 | -1.2 (1.3) | 8.1 (9.3) | -1.1 (-1.5 to -0.7) | 8.0 (5.4 to 10.6) |
| **STP^e^** |  |  |  |  |  |
| **Mean trajectory** |  |  |  |  |  |
| Baseline | 55 | 4.2 (1.0) | 57.7 (10.2) | - | - |
| Follow-up at 6 months | 49 | 3.3 (1.0) | 60.7 (11.0) | - | - |
| Follow-up at 12 months | 38 | 3.0 (1.0) | 61.8 (12.2) | - | - |
| Follow-up at 18 months | 22 | 3.3 (1.2) | 63.4 (12.8) | - | - |
| Follow-up at 24 months | 16 | 2.8 (1.2) | 67.9 (13.4) | - | - |
| **Change from baseline** Change from baseline |  |  |  |  |  |
| 6 months vs baseline | 49 | -0.9 (1.0) | 2.9 (6.4) | -0.9 (-1.2 to -0.6) | 3.3 (1.4 to 5.2) |
| 12 months vs baseline | 38 | -1.2 (1.0) | 5.1 (6.3) | -1.2 (-1.5 to -0.9) | 5.1 (3.1 to 7.2) |
| 18 months vs baseline | 22 | -1.0 (1.1) | 6.0 (7.1) | -0.9 (-1.3 to -0.5) | 5.6 (3.0 to 8.1) |
| 24 months vs baseline | 16 | -1.4 (1.3) | 9.5 (11.3) | -1.4 (-1.8 to -0.9) | 8.1 (5.2 to 11.0) |
| ATP vs STP, difference at baseline | - | - | \| - \| \| --- \| \| - \| \| - \| \| - \| \| - \| | -0.3 (-0.6 to 0.1) | 0.8 (-2.4 to 3.9) |
| ATP vs STP, difference at follow-up at 6 months | - | - | - | -0.1 (-0.4 to 0.3) | 0.1 (-3.1 to 3.4) |
| ATP vs STP, difference at follow-up at 12 months | - | - | - | 0.1 (-0.3 to 0.6) | 0.4 (-3.0 to 3.8) |
| ATP vs STP, difference at follow-up at 18 months | - | - | - | -0.5 (-1.0 to 0.03) | 2.6 (-1.4 to 6.6) |
| ATP vs STP, difference at follow-up at 24 months | - | - | - | -0.02 (-0.6 to 0.6) | 0.6 (-3.7 to 5.0) |
| ATP vs STP, difference in follow-up at 6 months vs baseline differences | - | - | - | 0.2 (-0.2 to 0.6) | -0.6 (-3.2 to 1.9) |
| ATP vs STP, difference in follow-up at 12 months vs baseline differences | - | - | - | 0.4 (-0.1 to 0.8) | -0.4 (-3.2 to 2.4) |
| ATP vs STP, difference in follow-up at 18 months vs baseline differences | - | - | - | -0.2 (-0.8 to 0.3) | 1.8 (-1.6 to 5.3) |
| ATP vs STP, difference in follow-up at 24 months vs baseline differences | - | - | - | 0.2 (-0.4 to 0.9) | -0.1 (-4.0 to 3.8) |

^a^CGI: Clinical Global Impression scale; severity of illness; range 1 to 7, higher is more severe.

^b^GAF: global assessment of functioning; range 0 to 100, higher is better functioning.

^c^From mixed-effects linear regression models adjusted for study site, consulting psychiatrist, and language of the interview, as well as clustering due to patient. The model for global assessment of functioning was further adjusted for clustering due to the referring physician.

^d^ATP: asynchronous telepsychiatry.

^e^STP: synchronous telepsychiatry.

# Table S4. Secondary outcomes: patient self-reported 12-Item Short Form Health Survey (physical and mental) scores at baseline and 6-, 12-, 18-, and 24-month follow-up for the 118 patients included in the secondary analysis.

| **Secondary outcomes** | **n** | **PHS-12^a^,**  **mean (SD)** | **MHS-12^b^, mean (SD)** | **PHS-12; estimate,**  **mean (95% CI)^c^** | **MHS-12; estimate,**  **mean (95% CI)^c^** | |
| --- | --- | --- | --- | --- | --- | --- |
| **ATP^d^** |  |  |  |  |  | |
| **Mean trajectory** rajectory |  |  |  |  |  | |
| Baseline | 52 | 39.6 (11.6) | 34.4 (9.6) | - | - | |
| Follow-up at 6 months | 51 | 39.5 (11.5) | 36.7 (9.8) | - | - | |
| Follow-up at 12 months | 42 | 38.7 (11.5) | 38.2 (9.1) | - | - | |
| Follow-up at 18 months | 28 | 41.1 (10.7) | 37.3 (8.5) | - | - | |
| Follow-up at 24 months | 21 | 40.7 (14.3) | 37.8 (11.9) | - | - | |
| **Change from baseline** Change from baseline |  |  |  |  |  | |
| 6 months vs baseline | 43 | -1.4 (8.8) | 2.0 (11.9) | -1.3 (-3.9 to 1.3) | 2.8 (-0.5 to 6.1) | |
| 12 months vs baseline | 33 | 0.3 (9.3) | 3.7 (12.5) | -0.4 (-3.3 to 2.6) | 4.0 (0.4 to 7.7) | |
| 18 months vs baseline | 22 | 0.3 (5.7) | 3.3 (10.5) | 0.3 (-3.1 to 3.7) | 3.3 (-1.0 to 7.5) | |
| 24 months vs baseline | 15 | 0.5 (7.6) | 4.6 (14.0) | 0.2 (-3.6 to 3.9) | 3.5 (-1.2 to 8.2) | |
| **STP^e^** |  |  |  |  | |  |
| **Mean trajectory** |  |  |  |  | |  |
| Baseline | 46 | 43.5 (10.3) | 31.7 (8.8) | - | | \| - \| \| --- \| \| - \| \| - \| \| - \| \| - \| |
| Follow-up at 6 months | 41 | 41.3 (10.5) | 36.0 (11.1) | - | | - |
| Follow-up at 12 months | 28 | 43.9 (9.4) | 34.3 (10.4) | - | | - |
| Follow-up at 18 months | 22 | 42.4 (10.6) | 38.1 (13.7) | - | | - |
| Follow-up at 24 months | 12 | 43.4 (6.4) | 36.3 (10.3) | - | | - |
| **Change from baseline** Change from baseline |  |  |  |  | |  |
| 6 months vs baseline | 34 | -1.8 (11.4) | 5.1 (10.4) | -2.2 (-5.0 to 0.6) | | 4.7 (1.2 to 8.2) |
| 12 months vs baseline | 24 | -1.1 (8.9) | 5.0 (9.9) | 0.3 (-2.9 to 3.4) | | 3.5 (-0.5 to 7.4) |
| 18 months vs baseline | 21 | -0.7 (8.4) | 5.2 (11.4) | -1.8 (-5.2 to 1.5) | | 6.0 (1.7 to 10.3) |
| 24 months vs baseline | 11 | -1.6 (11.6) | 2.2 (8.5) | -2.7 (-7.0 to 1.6) | | 4.1 (-1.4 to 9.6) |
| ATP vs STP, difference at baseline | - | - | - | -15.5 (-36.6 to 5.5) | | -5.0 (-22.9 to 12.8) |
| ATP vs STP, difference at follow-up at 6 months | - | - | - | -14.6 (-35.7 to 6.4) | | -7.0 (-24.7 to 10.7) |
| ATP vs STP, difference at follow-up at 12 months | - | - | - | -16.2 (-37.3 to 5.0) | | -4.5 (-22.4 to 13.5) |
| ATP vs STP, difference at follow-up at 18 months | - | - | - | -13.4 (-34.5 to 7.7) | | -7.8 (-25.6 to 10.1) |
| ATP vs STP, difference at follow-up at 24 months | - | - | - | -12.7 (-33.8 to 8.5) | | -5.6 (-23.6 to 12.4) |
| ATP vs STP, difference in follow-up at 6 months vs baseline differences | - | - | - | 0.9 (-2.9 to 4.7) | | -2.0 (-6.8 to 2.9) |
| ATP vs STP, difference in follow-up at 12 months vs baseline differences | - | - | - | -0.6 (-4.9 to 3.7) | | 0.6 (-4.8 to 6.0) |
| ATP vs STP, difference in follow-up at 18 months vs baseline differences | - | - | - | 2.1 (-2.7 to 7.0) | | -2.7 (-8.7 to 3.3) |
| ATP vs STP, difference in follow-up at 24 months vs baseline differences | - | - | - | 2.9 (-2.9 to 8.6) | | -0.6 (-7.8 to 6.7) |

^a^PHS-12: 12-item short form health survey physical health summary score; range 0 to 100, higher is better physical health.

^b^MHS-12: 12-item short form health survey mental health summary score; range 0 to 100, higher is better mental health.

^c^From mixed-effects regression models adjusted for study site, consulting psychiatrist, and language of the interview, as well as clustering due to patient and primary care physician.

^d^ATP: asynchronous telepsychiatry.

^e^STP: synchronous telepsychiatry.

# Table S5. Secondary outcomes: patient self-reported Patient Health Questionnaire-9 scores at baseline and 6-, 12-, 18-, and 24-month follow-up for the 118 patients included in the secondary analysis.

| **Secondary outcomes** | **n** | **PHQ-9^a^*,***  **mean (SD)** | **PHQ-9; estimate,**  **mean (95% CI)^b^** |
| --- | --- | --- | --- |
| **ATP^c^** |  |  |  |
| **Mean trajectory** |  |  |  |
| Baseline | 61 | 12.4 (7.2) | - |
| Follow-up at 6 months | 57 | 9.8 (6.7) | - |
| Follow-up at 12 months | 45 | 10.0 (6.0) | - |
| Follow-up at 18 months | 28 | 8.1 (5.6) | - |
| Follow-up at 24 months | 21 | 7.6 (5.7) | - |
| **Change from baseline** |  |  |  |
| 6 months vs baseline | 55 | -2.3 (4.4) | -2.5 (-3.9 to -1.1) |
| 12 months vs baseline | 43 | -2.8 (5.2) | -2.4 (-4.0 to -0.8) |
| 18 months vs baseline | 26 | -2.7 (3.8) | -2.8 (-4.7 to -0.8) |
| 24 months vs baseline | 19 | -2.6 (4.2) | -2.5 (-4.6 to -0.4) |
| **STP^d^** |  |  |  |
| **Mean trajectory** |  |  |  |
| Baseline | 54 | 12.6 (6.8) | - |
| Follow-up at 6 months | 40 | 10.8 (6.5) | - |
| Follow-up at 12 months | 34 | 11.9 (7.1) | - |
| Follow-up at 18 months | 22 | 10.5 (7.4) | - |
| Follow-up at 24 months | 17 | 9.2 (6.6) | - |
| **Change from baseline** |  |  |  |
| 6 months vs baseline | 40 | -0.7 (4.8) | -1.0 (-2.5 to 0.6) |
| 12 months vs baseline | 33 | -0.5 (6.4) | -0.7 (-2.4 to 0.9) |
| 18 months vs baseline | 22 | -3.0 (5.1) | -2.4 (-4.4 to -0.5) |
| 24 months vs baseline | 16 | -2.7 (6.8) | -3.4 (-5.6 to -1.2) |
| ATP vs STP, difference at baseline | - | - | -0.9 (-11.3 to 9.5) |
| ATP vs STP, difference at follow-up at 6 months | - | - | -2.4 (-12.8 to 8.0) |
| ATP vs STP, difference at follow-up at 12 months | - | - | -2.6 (-13.0 to 7.9) |
| ATP vs STP, difference at follow-up at 18 months | - | - | -1.2 (-11.7 to 9.2) |
| ATP vs STP, difference at follow-up at 24 months | - | - | 0.04 (-10.5 to 10.5) |
| ATP vs STP, difference in follow-up at 6 months vs baseline differences | - | - | -1.5 (-3.6 to 0.6) |
| ATP vs STP, difference in follow-up at 12 months vs baseline differences | - | - | -1.7 (-4.0 to 0.6) |
| ATP vs STP, difference in follow-up at 18 months vs baseline differences | - | - | -0.3 (-3.1 to 2.4) |
| ATP vs STP, difference in follow-up at 24 months vs baseline differences | - | - | 0.9 (-2.1 to 4.0) |

^a^PHQ-9: Patient Health Questionnaire-9; range 0 to 27, higher is more depressed.

^b^From mixed-effects regression models adjusted for study site, consulting psychiatrist, and language of the interview, as well as clustering due to patient and primary care physician.

^c^ATP: asynchronous telepsychiatry.

^d^STP: synchronous telepsychiatry.

# Figure S1. Participants flow through 24-month follow-up. ATP: asynchronous telepsychiatry; STP: synchronous telepsychiatry.


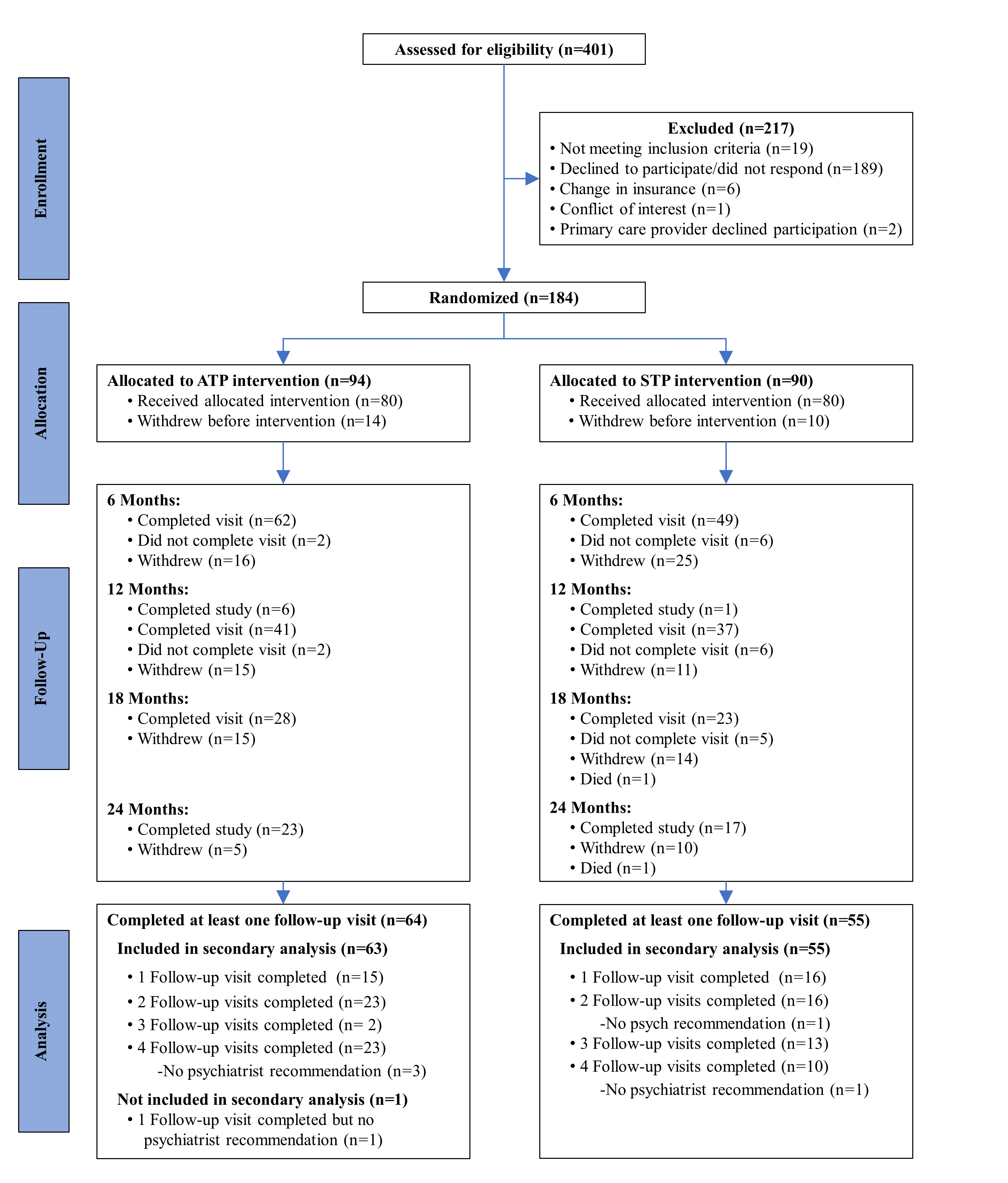

Supplement: Multimedia Appendix 1 [file jmir_v23i7e24047_app1.docx]
